# Finite Dimension: A Mathematical Tool to Analise Glycans

**DOI:** 10.1038/s41598-018-22575-4

**Published:** 2018-03-13

**Authors:** J. M. Alonso, A. Arroyuelo, P. G. Garay, O. A. Martin, J. A. Vila

**Affiliations:** 1 IMASL-CONICET, Universidad Nacional de San Luis, Ejército de Los Andes 950, 5700 San Luis, Argentina; 2 Universidad Nacional de Cuyo, Padre Jorge Contreras 1300, M5502JMA Mendoza, Argentina

## Abstract

There is a need to develop widely applicable tools to understand glycan organization, diversity and structure. We present a graph-theoretical study of a large sample of glycans in terms of *finite dimension*, a new metric which is an adaptation to finite sets of the classical Hausdorff “fractal” dimension. Every glycan in the sample is encoded, via finite dimension, as a point of *Glycan Space*, a new notion introduced in this paper. Two major outcomes were found: (a) the existence of universal bounds that restrict the universe of possible glycans and show, for instance, that the graphs of glycans are a *very* special type of chemical graph, and (b) how Glycan Space is related to biological domains associated to the analysed glycans. In addition, we discuss briefly how this encoding may help to improve search in glycan databases.

## Introduction

Of the four main biomolecular groups: proteins, nucleic acids, lipids and glycans, the latter has a greater structural complexity associated with their molecular diversity^1^. This is a consequence of the high number of different monomeric units, the number of different available linkages between these units, and their capability of branching.

Our research was motivated by basic questions about glycan structure: Are (the graphs of) glycans special in some way? Are they different from general chemical graphs? If so, in which way? Are glycans that appear exclusively in Bacteria and those that appear exxclusively in Eukaryota, significantly different? To answer these, we consider a large sample of glycans and strip them of all information, except for the underlying mathematical graph. When considered as mathematical graphs, glycans can be described as trees–branched or linear–and, less frequently, cyclic graphs^2^. We then use *finite dimension* to map this sample to Glycan Space, a subset of the plane we introduce here. This map answers the questions above and raises new, interesting questions of biochemical relevance. We study horizontal lines in Glycan Space in Section (3.4.1), and give proofs in an Appendix (see Supplementary Information).

This is, as far as we know, a new, pioneer application of finite dimension to the study of glycans. The novelty lies in the use of finite dimension, not of graphs, which have certainly been used previously, see for instance the work of^3,4^ applying graph theory to glycan databases, mainly for data-mining significant subtrees or motifs from glycans.

## Methods

Most of the glycans we study are entries in *GlyTouCan* (GTC), a large open database containing, at present (retrieved in April 2017), around 82,000 glycans, see^5^. GTC incorporates many other databases, including GlycomeDB, Carbbank(CCSD), GLYCOSCIENCES.de, PubChem CID, and UniCarbKB. The datasets analysed during the current study are available at: https://github.com/BIOS-IMASL/finite-dimension-for-glycan-analysis.

**Figure 1:**
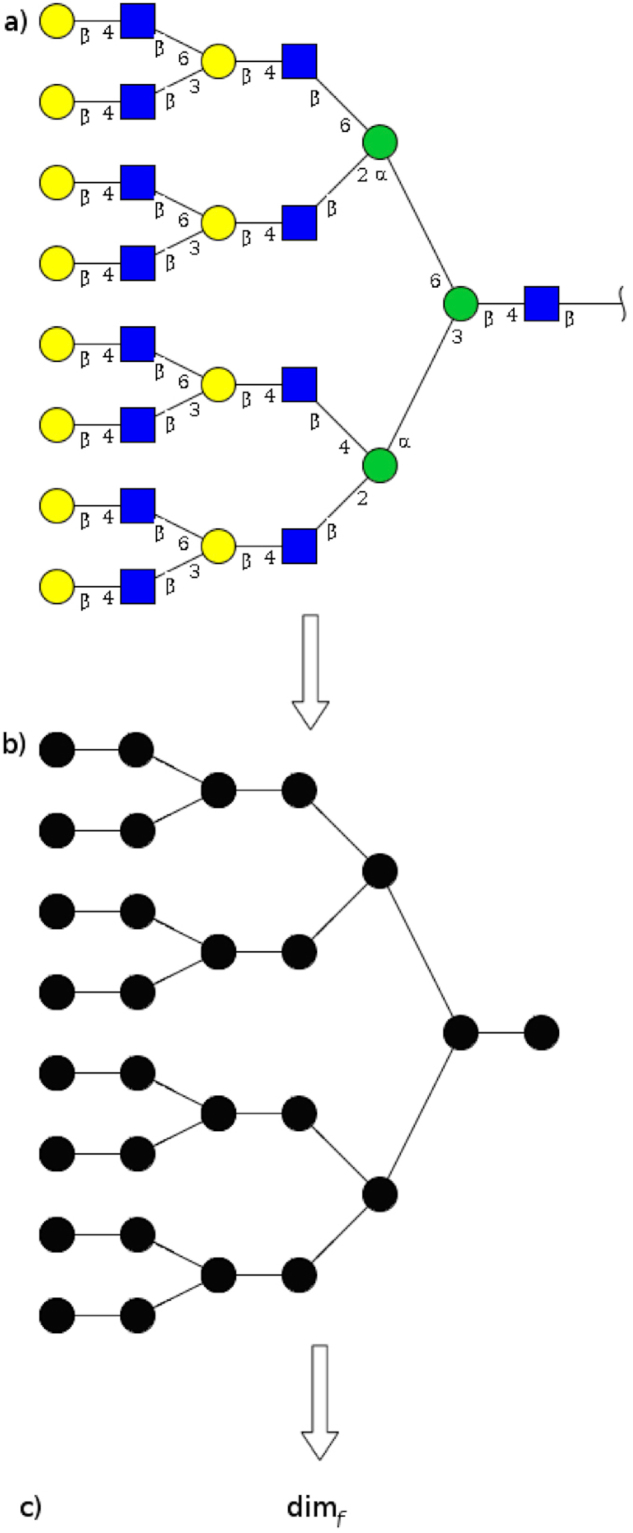
The sequence in (1) exemplified with an entry of GlyTouCan. (**a**) GTC Accession Number G06222QR, where yellow and green circles, and blue squares, represent different types of monosaccharides, Greek letters indicate the type of glycosidic bond, and numbers indicate the carbons involved in each bond; (**b**) Unlabeled graph representation of (a), where vertices represent monosaccharides, and edges, the connecting bonds; (**c**) the dim_*f*_ of graph (b) which, in this particular example, is ${{\rm{\dim }}}_{f}(G06222QR)=\,\mathrm{ln}\,15/\mathrm{ln}\,10\sim 1.17609$.

Part of the information contained in a typical entry of GTC is an underlying graph which is finite, simple, undirected and connected (Fig. 1a). We remove all chemical information to obtain a graph (Fig. 1b), compute the finite dimension (denoted dim_*f*_) of the graph, and call it the *finite dimension of the glycan* in question. Schematically (see Fig. 1):

1 $${\rm{glycan}}\mapsto {\rm{\Gamma }}({\rm{glycan}})\mapsto {{\rm{\dim }}}_{f}({\rm{\Gamma }}({\rm{glycan}}))\,:={{\rm{\dim }}}_{f}({\rm{glycan}}),$$

where Γ(glycan) denotes the underlying graph (Fig. 1b), and ${{\rm{\dim }}}_{f}({\rm{\Gamma }}({\rm{glycan}}))$ is the finite dimension of this graph. The finite dimension of glycan is defined by the last equality in (1). In line with this definition, we abuse language and apply directly to glycans notions that are graph-theoretical, for example we say that two glycans are isomorphic when their associated graphs are isomorphic.

### Finite dimension

In this section we collect the definition and some facts about finite dimension; for more information consult^6,7^. Recently introduced, this dimension is an adaptation to finite sets of the classical Hausdorff (or “fractal”) dimension^8^, see^9^ for a modern treatment.

The Hausdorff dimension of finite sets is zero. In contrast, the finite dimension of finite sets is highly non-trivial, making it suitable to classify finite sets. We call it *finite* dimension because it is defined *only* on finite sets; its values, however, can be *any* real number ≥0, or infinity^6^.

Finite dimension is actually defined on finite metric spaces (for the general definition and properties see^6^). Graphs can be used–in several ways^7^–to give a metric structure to the set of vertices. Here we use the simplest way, standard in Graph Theory, which is to count the smallest number of hops between two vertices, using only adjacent vertices to go from one to the other. The finite dimension of this metric space is called the finite dimension of the graph and, in this paper, of the glycan in question. An important fact is that, with this metric, isomorphic graphs have equal finite dimension^7^.

In the graphs obtained from GTC most edges have the same length, but ramified glycans contain chemical ligatures whose length is approximately 1.5 times the common, “usual”, length (e.g. 1–6 linkages). We disregard this difference and assume that all edges have length one. This has an important technical implication that simplifies the calculation of dim_*f*_. In fact, if Γ denotes a graph with vertices *V*, we have:

2 $${{\rm{\dim }}}_{f}({\rm{\Gamma }})=\frac{\mathrm{ln}(N)}{\mathrm{ln}(D)}=\frac{\mathrm{ln}(\vartheta )}{\mathrm{ln}(D)}$$

where *N* is the smallest number of *cliques* (i.e. sets of vertices of diameter 1) that are needed to cover *V*, and *D* is the diameter of Γ. It turns out that *N* = *ϑ*(Γ), a classical graph parameter called *clique covering number*. For *perfect graphs*, Lovász has shown that *ϑ*(Γ) = *α*(Γ), the *independence number* of Γ^10,11^. The vast majority of the glycans we study are trees, a class of perfect graphs. Hence:

$${{\rm{\dim }}}_{f}({\rm{\Gamma }})=\frac{\mathrm{ln}(\alpha ({\rm{\Gamma }}))}{\mathrm{ln}(D)}$$

This is the formula used in the paper to compute finite dimension. In doing so, we use the open-source mathematical software system SAGEMATH^12^.

#### Notation and examples

In this paper, *graph* means finite, undirected, simple and connected graph. The *path P*_*n*_ has *n* vertices and *n* − 1 edges; it has diameter *n* − 1. The *star St*_*n*_ has *n* vertices, with a central one which is adjacent to all other vertices; it has diameter 2. Let *C*_*n*_ denote the cycle with *n* vertices and *n* edges; it has diameter $\lfloor n\mathrm{/2}\rfloor$, where $\lfloor n\mathrm{/2}\rfloor$ is the largest integer ≤*n*/2. The complete graph on *n* ≥ 2 vertices is denoted *K*_*n*_; its diameter is 1, for all *n*. The finite dimension of a graph is zero if and only if (iff) the graph is a single point, and ${{\rm{\dim }}}_{f}({\rm{\Gamma }})=\infty$ iff Γ = *K*_*n*_, for some *n* ≥ 2. For all other graphs, dim_*f*_ is a positive real number. If Γ is *triangle-free* the cliques used to cover *V*(Γ) are pairs of adjacent vertices and, hence, *n* ≤ 2*N*, or $\lceil n\mathrm{/2}\rceil \le N$, where $\lceil n\mathrm{/2}\rceil$ denotes the smallest integer ≥*n*/2.

## Results

We study a sample ${\mathscr{S}}$ that consists of two different sets: a portion of the glycan database GTC, and a set of “synthetic” glycogen containing 500 simulated glycogen molecules. The complete sample ${\mathscr{S}}$ is available online (see Supplementary information below).

### The contents of GlyTouCan

We study the contents of GTC from the point of view of the graphs associated to each entry. We read all entries of GTC with WURCS codes^13^, and considered alternative linkage information (alternative, statistically or range), but we did not consider alternative units or alternative repetitions. We obtained 52,374 entries, 25 of which were discarded as they were disconnected. The remaining set of 52,349 connected graphs, denoted *gtc*, is the *disjoint* union of a set *T* that contains only trees and a set *Cy* of graphs that are not trees, i.e. graphs that are, or contain, cycles. In its turn, *T* is the disjoint union (denoted $\coprod$) of *B*, the set of *branched* or *ramified* trees, and *L*, the set of *linear* trees, i.e. paths of different lengths. We have:

$$gtc=T\,\coprod \,Cy=B\,\coprod \,L\,\coprod \,Cy.$$

The size of these sets, and their percentage in *gtc*, is: |*B*| = 28, 424 (54.4%), |*L*| = 23, 715 (45.3%), and |*Cy*| = 210 (0.4%). We can further subdivide *Cy* as the disjoint union of *C* and *Cb*, where *C* denotes the set of graphs that are pure cycles, and *Cb* the set of ramified ones, i.e. the graphs that *contain*, but are not themselves, cycles. We have |*C*| = 130 (61.9%) and |*Cb*| = 80 (38.1%).

Some of the entries in GTC contain information about the biological species where their associated glycan was found. We follow^14^ and group each of these species into three domains: *Eukaryota*, denoted *EU*, *Bacteria*, *BA*, and *Archaea*, *AR*. We could read 15,230 glycan structures included in this taxonomy. Of these, 876 entries were not uniquely categorized, and were removed from *tax*, the set of entries uniquely classified. We have:

$$tax=EU\,\coprod \,BA\,\coprod \,AR,$$

with |*tax*| = 14, 354, |*EU*| = 8, 411 (58.6%), |*BA*| = 5, 901(41.1%), and |*AR*| = 42(0.3%). Unfortunately, not all elements of *tax* are in *gtc*. We study only those that are part of *gtc*, and use the following notation: *EUB* for the set of branched eukaryotic glycans, *EUL* the set of linear ones, and *EUC* for those eukaryotes that are, or contain, cycles, which turns out to be empty. Similarly, *BAB* denotes the set of branched bacteria, etc. The sizes are: |*EUB*| = 4, 589 (59.4%), where the percentage in parenthesis refers to the proportion of branched eukaryotic glycans in the total number of eukaryotic glycans, and similarly for the other sets; thus, |*EUL*| = 3, 138 (40.6%), and |*EUC*| = 0 (0.0%). For bacteria, we have |*BAB*| = 1, 631 (51.1%), |*BAL*| = 1, 561 (48.9%) and |*BAC*| = 2(0.06%). Finally, for archaea, |*ARB*| = 3 (8.3%), |*ARL*| = 33 (91.7%) and |*ARC*| = 0 (0.0%). We summarize these facts in Table 1 below.

**Table 1:** Contents of GlyTouCan classified in terms of the 3 biological domains: Eukaryota (EU), Bacteria (BA) and Archaea (AR), and in terms of their graph properties: branched (B), linear (L) and cyclic or containing cycles (Cy).

		gtc			GTC	
		B	L	Cy		
		22 201	18 983	208		
tax	EU	4 589	3 138	0	684	8 411
	BA	1 631	1 561	2	2 707	5 901
	AR	3	33	0	6	42
		6 223	4 732	2		

Table 1 consists of three rectangles. One is called *gtc* and consists of the three columns *B*, *L*, *Cy*. The second rectangle is called *tax* and consists of three rows labelled *EU*, *BA*, *AR*, and six columns. The third rectangle is labelled *GTC* and consists of four columns (*B*, *L*, *Cy*, *GTC*) and four rows. The main portion of the table consists of the 9 entries defined by *EU*, *BA*, *AR* and *B*, *L*, *Cy*. For instance, there are 4,589 branched eukaryotic glycans and 3,138 linear ones.

Outside of the table proper there is a column and a row: 8,411 for instance, is the sum of all elements of *EU*, including 684 eukaryotic glycans that belong to GTC but not to *gtc*, etc. Similarly, 6,223 is the amount of branched glycans that are categorized, i.e. that belong to *tax*. The number 22,201 in the cell labelled *B* gives the number of branched elements of *gtc* that are not categorized, i.e. that do not belong to *tax*, and similarly for the cells labelled *L*,*Cy*.

#### Branched glycans in *gtc*

The set of dimensions of elements of *B* is denoted dimB, and its statistical structure is summarised in Tables 2 and 3. It follows that $0.7737\le {{\rm{\dim }}}_{f}\le 2.0$, for *all* ramified glycans in *gtc*. We can also note that dimB has only 115 *different* values.

**Table 2:** Summary of the finite dimensions of glycans in ${\mathscr{S}}$, where dimB, dimL, dimC and dimCb stand, respectively, for the sets of finite dimensions of elements of B, L, C and Cb, and S500 stands for the set of finite dimensions of synthetic glycogen (see Subsection 3.2).

Sets of glycans	Min.	1st Qu.	Median	Mean	3rd Qu.	Max.
dimB	0.7737	0.8982	1.0	1.0140	1.0570	2.0
S500	0.9306	0.9850	1.0180	1.0350	1.0680	1.2680
dimL	0.6309	0.6309	0.7925	0.8137	1.0	1.0
dimC	1.0	1.0	1.262	1.184	1.262	1.585
dimCb	0.8271	0.8982	1.0	0.9685	1.0	1.1610

**Table 3:** Deciles of dimB and dimL.

Glycans	10%	20%	30%	40%	50%	60%	70%	80%	90%
dimB	0.8368	8.6170	0.9208	1.000	1.000	1.000	1.000	1.086	1.1606
dimL	0.6309	0.6309	0.6309	0.7324	0.7924	0.7924	1.0	1.0	1.0

#### Linear glycans in *gtc*

There are 23,715 linear glycans, i.e. paths *P*_*n*_, in *gtc*. Of these, 4,015 are segments *P*_2_. In general, many non-isomorphic graphs can have the same finite dimension, but linear graphs have a very simple structure: two paths *P*_*n*_, *P*_*m*_, are isomorphic iff *n* = *m*, iff *P*_*n*_ and *P*_*m*_ have the same diameter, iff ${{\rm{\dim }}}_{f}({P}_{n})={{\rm{\dim }}}_{f}({P}_{m})$. In particular, their finite dimension depends only on the diameter of the path. Indeed, ${{\rm{\dim }}}_{f}({P}_{2})=\infty$ and, for *n* ≥ 3, it is given by ${{\rm{\dim }}}_{f}({P}_{n})=\,\mathrm{ln}(\lceil n\mathrm{/2}\rceil )/\mathrm{ln}(n-\mathrm{1)}$. These dimensions are always <1, except for the case *n* = 3, where it equals 1. On the other hand, their limit (as *n* → ∞) is 1. We note here that there are 24 different values of the finite dimension of linear glycans in *gtc*, 23 finite ones and infinity. Thus, the total of 23,715 linear glycans falls into only 24 different isomorphism classes.

**Figure 2:**
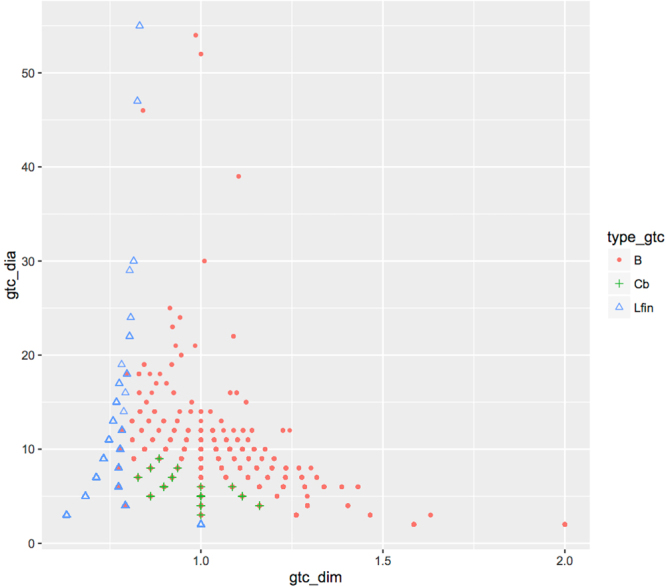
Representation of *gtc* in Glycan Space, distinguishing glycans that are branched trees (red dots), branched cyclic (green crosses), and linear with ≥2 edges (blue triangles).

**Figure 3:**
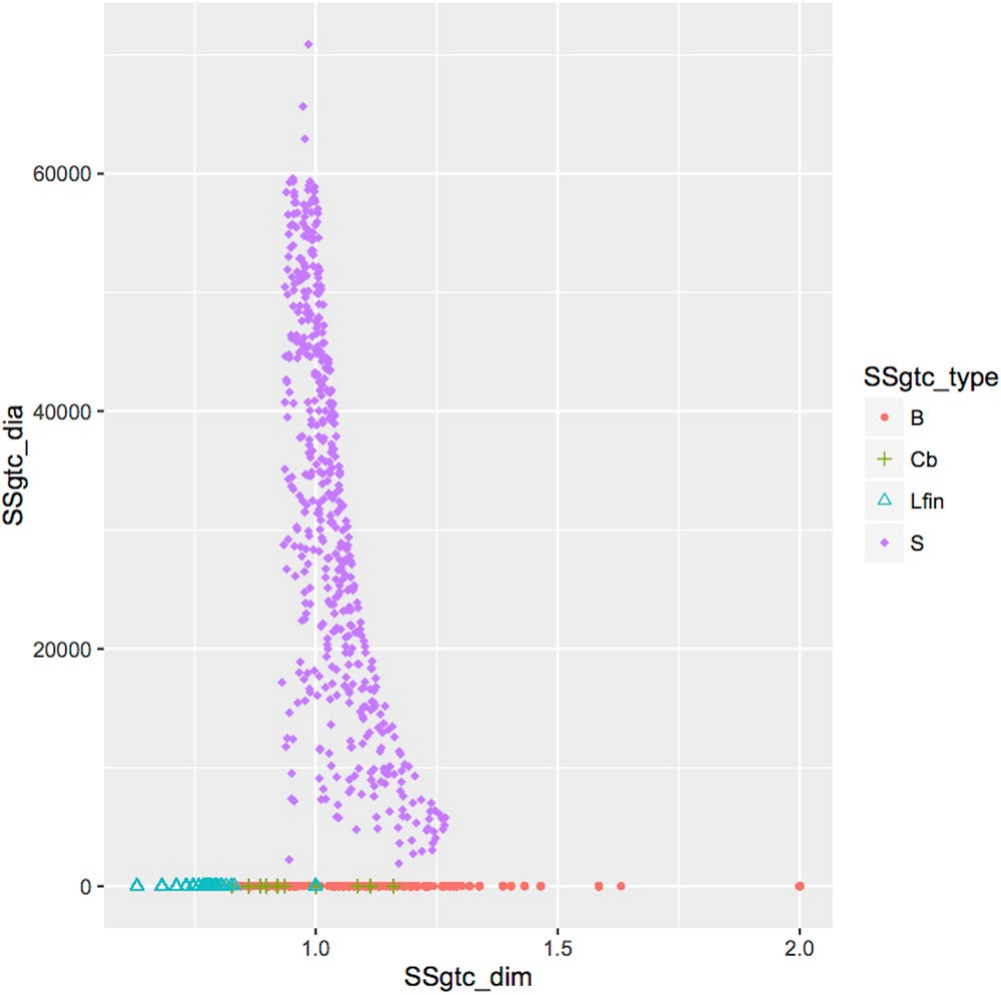
Representation of *gtc* (at the bottom, using similar colors and symbols as in Fig. 2) together with *S500* (purple rhombuses) in Glycan Space.

Since the finite dimension of linear glycans gives no more information than their diameter, it may seem that considering dim_*f*_ only complicates matters unnecessarily. However, there are advantages to treat all glycans uniformly, the most important of which is to discover the unexpected way in which the linear glycans fit in $\gamma (gtc),\,\gamma ({\mathscr{S}})$ (cf. Section 3.5 and Figs 2 and 3).

We let *Lfin* denote the 19,700 glycans that are paths of length ≥2, and let dimL denote the set of their finite dimensions. From Table 3 we can read, for example, that more than 30% of the linear glycans of length ≥2 consists of paths of length 2 (because *P*_3_ is the *only* path with dim_*f*_ = 1).

#### Cyclic glycans in *gtc*

Four out of the 130 glycans in *C* are triangles *C*_3_, and their finite dimension is infinity. Note that these four *C*_3_’s are the *only* glycans of *gtc* that are not triangle-free. We let dimC denote the set of 126 purely cyclic glycans of *finite* dim_*f*_, i.e. *C*_*n*_ for *n* ≥ 4. In dimC there are exactly 3 different values: 1.0, 1.1609 and 1.5849. We see that all these values are ≥1. We have ${{\rm{\dim }}}_{f}({C}_{3})=\infty$, and ${{\rm{\dim }}}_{f}({C}_{n})=\,\mathrm{ln}(\lceil n\mathrm{/2}\rceil )/\mathrm{ln}(\lfloor n\mathrm{/2}\rfloor )$ for *n* ≥ 4. It follows that ${{\rm{\dim }}}_{f}({C}_{n})=1$ for *n* even, and >1 for *n* odd. Note also that ${{\rm{\dim }}}_{f}({C}_{2k+1})\to 1$, when *k* → ∞. In contrast to the case of paths and pure cycles, the values in dimCb lie on both sides of 1. There are 80 elements in *Cb*, and 11 different values in dimCb.

### Glycogen

Using NumPy^15^ we generated 500 synthetic graphs that satisfy the following specification: the graphs are ramified trees with up to 120,000 nodes, and branches of length 20–23 nodes that sprout every 12–19 nodes^16,17^. We let S500 denote the set of their finite dimensions (see Table 2).

### Universal bounds

From Table 2 one can read universal bounds $a\le {{\rm{\dim }}}_{f}\le b$ for the different types of glycans of sample ${\mathscr{S}}$. For instance, the choice *a* = 0.6309, *b* = 2.0, works for ${\mathscr{S}}$ and *a* = 0.9341, *b* = 1.2680, for *S500*. By Equation (2), the inequalities $a\le {{\rm{\dim }}}_{f}({\rm{\Gamma }})\le b$, are equivalent to:

3 $${D}^{a}\le N\le {D}^{b}.$$

Since both *N* and *D* can be regarded as different measures of size for Γ, Equation (3) establishes relations between these measures that restrict, both qualitatively and quantitatively, Γ’s form and, ultimately, the kind of graphs that can pertain to glycans. More on this in Section 3.4.

### Glycan Space

We enrich the information provided by the finite dimension of glycans by adding information on size, in the form of the glycan’s diameter. We feel this is an appropriate way to compactly codify glycan information, or rather, the glycan information that is the focus of this paper. To this effect, we introduce *Glycan Space* (${\mathscr{G}}{\mathscr{S}}$), a subset of the plane ${{\mathbb{R}}}^{2}$ where all glycans of ${\mathscr{S}}$ can be represented or *coded*. In fact, our methods are so general that all glycans in any future sample will have a coding in ${\mathscr{G}}{\mathscr{S}}$, as long as we can obtain their underlying graphs. Consider the lattice ${\mathcal L} \subseteq {{\mathbb{R}}}^{2}$ of all points (*n*, *m*) with *n*, *m* integers ≥2, and let $\phi : {\mathcal L} \to {{\mathbb{R}}}^{2}$ be given by: *φ*(*n*, *m*) := (ln(*n*)/ln(*m*), *m*). By definition ${\mathscr{G}}{\mathscr{S}}\,:=\phi ( {\mathcal L} )$, the image of *φ*. Every graph Γ has a coding in ${\mathscr{G}}{\mathscr{S}}$, namely the point $({{\rm{\dim }}}_{f}({\rm{\Gamma }}),{\rm{diameter}}({\rm{\Gamma }}))$, but not all points of ${\mathscr{G}}{\mathscr{S}}$ are the code of a graph. For example, no graph has code *φ*(2, 10) (for a proof see (**A1**) of the Appendix in Supplementary Information).

The coding of ${\mathscr{S}}$ is defined to be $\gamma ({\mathscr{S}})$, where $\gamma :{\mathscr{S}}\to {\mathscr{G}}{\mathscr{S}}$,

$$\gamma (g)\,:=\,({{\rm{\dim }}}_{f}\,({\rm{\Gamma }}(g)),\,{\rm{diameter}}\,({\rm{\Gamma }}(g))).$$

Since ${\mathscr{S}}=gtc\cup S500$, we can restrict $\gamma$ to *gtc* or to *S*500, to obtain codings of these sub-samples. Figure 2 shows $\gamma (gtc)$, the coding of *gtc*, and a neat structure in it. More precisely, Fig. 2 includes *B*, *Cb* and *Lfin*. This set of 52,219 glycans is coded in ${\mathscr{G}}{\mathscr{S}}$ using 145 points.

#### Horizontal lines in ${\mathscr{G}}{\mathscr{S}}$

Let *D* ≥ 2, and consider the horizontal line in ${\mathscr{G}}{\mathscr{S}}$ defined by *D*, or *D*-line for short, i.e. the set of points of ${\mathscr{G}}{\mathscr{S}}$ whose second coordinate equals *D*. We are interested in obtaining information about the endpoints *L*_*D*_, *R*_*D*_ of *D*-lines when we restrict them to specific classes of graphs. Since the second coordinate of these points is fixed to *D*, we abuse notation and let *L*_*D*_, *R*_*D*_ denote both the points of the plane, and their corresponding first coordinates. Note also that the actual values of *L*_*D*_, *R*_*D*_ depend crucially on the class of graphs under consideration. We have:

1. For *D*-lines of triangle-free graphs, the leftmost point *L*_*D*_ coincides with the code of the path *P*_*D*+1_. If the graphs contain triangles (and *D* ≥ 3), then *L*_*D*_ = ln2/ln3, as shown in Theorem 5.2 of ^9^. The rightmost point *R*_*D*_ *(for arbitrary graphs)* does not exist: there are graphs of diameter *D* whose finite dimension is as large as desired. For a proof, see (**A2**) of the Appendix.
2. For *D*-lines of chemical graphs (i.e. *graphs whose vertices have degree* ≤4) *R*_*D*_ is finite, but tends to infinity with *D*. See (**A3**) of the Appendix.

Summarising, for a graph *g* of diameter *D*, we have:

$${L}_{D}\le {{\rm{\dim }}}_{f}(g)\le {R}_{D},$$

where *L*_*R*_ = ln2/ln3, *R*_*D*_ = ∞, if *g* is arbitrary, and ${L}_{D}=\,\mathrm{ln}(\lceil (D+1)/2\rceil )/\mathrm{ln}(D)$, *R*_*D*_ < ∞, but *R*_*D*_ → ∞ as D → ∞, if *g* is trian*g*le-free and chemical. The last case applies notably to glycans, since the vast majority of them are triangle-free, chemical graphs. But for glycans, we already know, from Table 3, that *R*_*D*_ ≤ 2. In the next section we show, moreover, that for $g\in gtc\cup {\mathscr{S}}$, *R*_*D*_ → 1 as _*D*_ → ∞. In other words, glycans are indeed a *very* special subset of the chemical graphs.

### The shape of γ ${\boldsymbol{(}}{\boldsymbol{g}}{\boldsymbol{t}}{\boldsymbol{c}}{\boldsymbol{)}}$ and γ ${\boldsymbol{(}}{\boldsymbol{S}}{\boldsymbol{)}}$

Figure 2 shows that $\gamma (gtc)$ has a shape that resembles that of a Christmas tree. In stark contrast to the general results of the last section, the rightmost bound of *D*-lines is always ≤2 and, moreover, tends to decrease as *D* grows. Since all elements of the sample ${\mathscr{S}}$ are triangle-free, we already knew that the left boundary of $\gamma (gtc)$ is given by the codes of paths. Figure 2 shows that glycans with *D* ≤ 20 come quite close to filling up the space to this theoretical boundary. Another interesting feature of $\gamma (gtc)$ is that, if you disregard the special case *D* = 2, and join the triangles coding the remaining linear glycans, you obtain two “lines” that get closer as *D* increases. Thus, the structural simplicity of linear glycans noted earlier, is reflected in ${\mathscr{G}}{\mathscr{S}}$ by the fact that they form a “1-dimensional” subset of the plane. In contrast, the far more complex ramified glycans form a “2-dimensional” pattern.

Figure 3 shows the coding of the complete sample ${\mathscr{S}}$. The large disparity in diameter between *gtc* and *S500* accounts for the fact that *gtc* appears completely flattened near *D* = 0. The Christmas tree pattern, however, remains unchanged. The reader may contrast the rich information contained in Figs 2 and 3 to the more classical statistical summaries of Table 2.

Next, we use Equation (3) to explain the apparent invariance of the Christmas tree shape. We study the left and right “curves” in ${\mathscr{G}}{\mathscr{S}}$ that delimit the region inside which $\gamma ({\mathscr{S}})$ lies. We start with another derivation and formulation of the leftmost boundary. Suppose that Γ is a ramified tree with *n* vertices and diameter *D*, and ${{\rm{\dim }}}_{f}({\rm{\Gamma }})\le b$, for 0 < *b* < 1 or, equivalently, *N* ≤ *D*^*b*^. Since Γ is ramified, *D* + 2 ≤ *n* and, since it is a tree, *n* ≤ 2*N*. Hence, *D* + 2 ≤ 2*D*^*b*^. We conclude that, conversely, *D* + 2 > 2*D*^*b*^ implies ${{\rm{\dim }}}_{f}(\Gamma ) > b$. For example, if *D* ≥ 20, then ${{\rm{\dim }}}_{f}({\rm{\Gamma }}) > 0.8$, or if *D* ≥ 1004, then ${{\rm{\dim }}}_{f}({\rm{\Gamma }}) > 0.9$. Clearly, as *D* increases, ${{\rm{\dim }}}_{f}({\rm{\Gamma }})$ increases to 1.

Similarly, if ${{\rm{\dim }}}_{f}({\rm{\Gamma }})\ge a$, for 1 < *a*, we have *D*^*a*^ ≤ *N*. The bound *N* ≤ *n* − 1, while not very sharp, is true for all graphs. Thus, *D*^*a*^ ≤ *n* − 1 or, roughly speaking, on the *D*-line, for dim_*f*_ to be “far” from 1 it is necessary to have a glycan with “many” nodes in a “small” space (i.e. the diameter must still equal *D*). Or, conversely, a glycan with diameter *D* and “few” nodes must have dim_*f*_ close to 1. For example, for *D* = 10 and *a* = 1.5, the above condition gives that *n* ≤ 32 implies ${{\rm{\dim }}}_{f}({\rm{\Gamma }}) < 1.5$. In actual fact, the rightmost point of $\gamma (gtc)$ on the 10-line corresponds to 6 graphs Γ with *n* = 28 (the largest *n* on the 10-line) and ${{\rm{\dim }}}_{f}({\rm{\Gamma }})=\,\mathrm{ln}\,(15)/\mathrm{ln}\,(10)\sim 1.17609$. It turns out that these 6 graphs are all isomorphic. The glycan with Accession Number G06222QR in GTC, shown in Fig. 1, is one such example: it has code $(1.17609,10)\in {\mathscr{G}}{\mathscr{S}}$. The implications of this condition for the rightmost boundary of $\gamma ({\mathscr{S}})$ is that existing glycans satisfy the following condition which summarises the qualitative and quantitative aspects discussed here and in subsections 3.3 and 3.4:

4 $$\begin{array}{c}glycans\,do\,not\,have\,a\,l{\arg }e\,number\,of\,nodes\\ in\,relation\,to\,the\,modecule\mbox{'}s\,diameter.\end{array}$$

As long as this condition remains true for glycans discovered in future, the Christmas tree shape will persist. The simulated molecules of glycogen are archetypicsal in relation to this property: they consist of a long path with lengths in the range 1,900–70,900, from which short paths ramify every so often.

In order to get a feeling for the meaning of dim_*f*_, we invite the reader to take a look at the glycans with Accesion Numbers G60741HS and G94498MI in GTC. Both have the same code $(0.77815,10)\in {\mathscr{G}}{\mathscr{S}}$, i.e. the leftmost point on the 10-line. There are exactly 22 such glycans in *B*, and only 2 isomorphism classes represented by the two glycans mentioned above. For comparison, there are 252 glycans in *B* with code $(1.0,10)\in {\mathscr{G}}{\mathscr{S}}$ (an intermediate point on the 10-line), which fall into 35 different isomorphism classes; glycans with Accession Numbers G18347PA and G24006CZ represent two examples out of the 35 classes.

**Figure 4:**
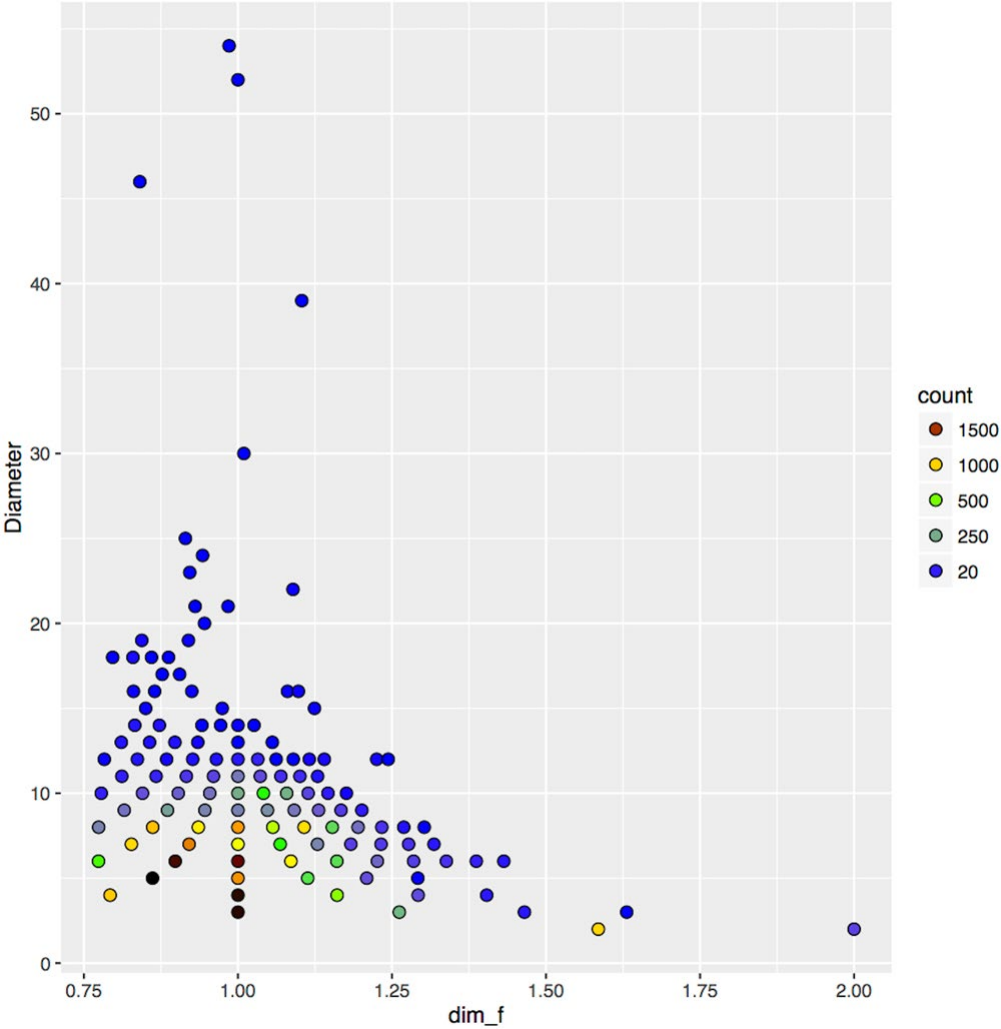
2D histogram of B in Glycan Space. Counts are shown in a color scale that goes from blue (lower count) through grey, green, yellow and red/brown (middle counts), to black (higher count).

Figure 4 is a 2D-histogram that shows, in a color scale, the number of glycans *g* ∈ *B* that have the same code $\gamma (g)\in {\mathscr{G}}{\mathscr{S}}$; this number varies between 1 and 1,994. The figure shows that most glycans in B have small diameter, say under 20. If we instead consider the number of isomorphism classes of glycans in *B* that have the same code, we obtain small figures in the range 1–63. This means that in *B* there are at most 63 different glycans (i.e. non-isomorphic glycans) with the same code. When we consider the total number of isomorphism classes of glycans in *B*, the figure |*B*| = 28,424 reduces to 1,343.

The information contained in Fig. 2 suggests several questions. For instance, the neat, regular pattern of point-lines ascending on both sides of the line dim_*f*_ = 1, is interrupted by a big hole for *D* in the range 15–20, and dim_*f*_ around 1 (as well as two smaller holes at about *D* = 14 or 15, on both sides of the line). “Should” there exist glycans filling up this gap? If so, what properties would they have? Can the known codes of the “missing” glycans give hints as to what or where to look for?

### Finite dimension and taxonomy

**Figure 5:**
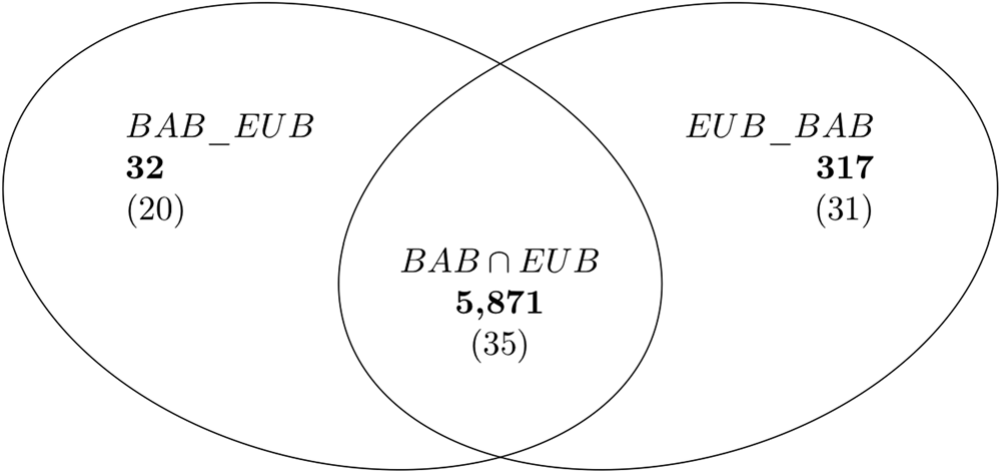
Exclusive and shared finite dimensions of *BAB* and *EUB*. Bold numbers indicate the total number of glycans in each set. Shown in parentheses is the number of different values of dim_*f*_ in each set.

We discuss the way in which the finite dimension of ramified tree glycans is related to glycan taxonomy. We use the notation *BAB* for branched *BA*, and *EUB* for branched *EU*, |*BAB*| = 1,631, and |*EUB*| = 4,589. We consider a sort of “symmetric difference” of these sets with respect to finite dimension. By definition, *BAB*_*EUB* consists of elements of *BAB* whose dim_*f*_ is *exclusive* for *BA*; similarly, *EUB*_*BAB* consists of glycans in *EUB* whose dim_*f*_ is not the finite dimension of any *BAB*. The sets contain, respectively, 32 and 317 glycans (bold figures in Fig. 5). The “intersection” consists of elements in the union *BAB*∪*EUB* (as sets) whose finite dimension is shared by *BA* and *EU*. The total number is, of course, 6,220. The set of *different* values of the finite dimension of these 6,220 glycans has a total of 86 elements, shown in parenthesis in Fig. 5, of which 20 are exclusive to *BA*, 31 exclusive to *EU*, and 35 are shared by *BA* and *EU*.

**Figure 6:**
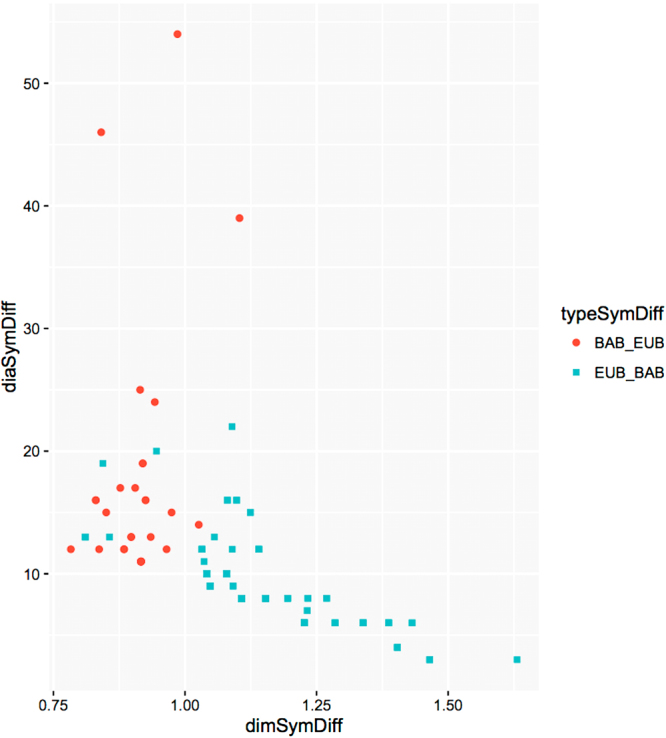
Codes in ${\mathscr{G}}{\mathscr{S}}$ of ramified tree glycans, from Bacteria in *BAB*_*EUB* (red circles) and from Eukaryota in *EUB*_*BAB* (cyan squares).

Figure 6 shows the position in ${\mathscr{G}}{\mathscr{S}}$ of the differences *BAB*_*EUB* and *EUB*_*BAB* of Fig. 5. We see a rather clear-cut separation between Bacteria and Eukaryota, as well as a shift to the right and down as we move from Bacteria to Eukaryota. In other words, glycans from Bacteria have “large” diameter and “small” finite dimension, and those from Eukaryota have “smaller” diameters and “large” dimension. This means, roughly speaking, “long and sparse” glycans for Bacteria, and “short and packed” (i.e. with many edges in the given diameter) glycans for Eukaryota.

Figure 6 suggests several questions; for instance, about exceptions and outliers. There are four exceptional *EUB*-points that have dim_*f*_ < 1. What can be said about the properties of glycans coded by these four points? And what about the *BAB*-points of diameters 14 and 39 that have dim_*f*_ > 1? Or glycans coded by the 3 points with diameter ≥35?

### Prospective uses of the glycan coding

An analysis of the advantages and disadvantages of existing methods to classify and retrieve glycan structures in databases is certainly out of the scope of this work. However, we foresee possible applications of the new methodology for this purpose, i.e., to search (by coding/decoding) a glycan database (DB) and, hence, we will refer briefly to this point.

Let us assume we wish to decide whether or not a glycan *g*, with underlying graph Γ(*g*), is an entry of a given DB. We start by encoding it as a point $\gamma (g)=P=({{\rm{\dim }}}_{f},dia)\in {\mathscr{G}}{\mathscr{S}}$. If *P* does not belong to the image $\gamma (DB)\subseteq {\mathscr{G}}{\mathscr{S}}$, we conclude that *g* is not in DB. If it does, we have to decode *P* and find a unique entry of DB that corresponds to *g*, provided one exists. To decode, the first step is to find out whether or not DB contains a graph Γ isomorphic to Γ(*g*), Γ ≅ Γ(*g*). It suffices to search through the isomorphism classes of graphs in DB. This is trivial if *g* is linear: we can just compute the length of Γ(*g*). To decode *P* when *g* is branched, recall that, of the 145 points of $\gamma (gtc)$, 18 are exclusive to linear glycans, so we concentrate on the 127 remaining ones. None of these points contain more than 63 isomorphic classes of graphs. Searching through these few classes we can decide very fast whether or not DB contains Γ ≅ Γ(*g*).

This suggests an algorithm to decide whether or not a given glycan *g* is in DB: we first compute its code $\gamma (g)$ and, if it is not in $\gamma (gtc)$, we conclude that *g* is not in DB. If the code is in $\gamma (gtc)$, we search for an isomorphic graph with this code (the largest such search-set contains 63 elements). If we cannot find an isomorphic graph, we conclude that *g* is not in the database. If we do find one, say Γ, then we search through all labelled graphs with Γ as underlying graph (the largest such search-set contains at most 1,994 elements) and again, if we find a labelled graph with the same labels as *g*, then *g* is in DB, and not otherwise.

## Conclusions and Questions

Via finite dimension we obtained a compact coding in ${\mathscr{G}}{\mathscr{S}}$ of the sample ${\mathscr{S}}$. The shape of $\gamma ({\mathscr{S}})\subset {\mathscr{G}}{\mathscr{S}}$ resembles that of a Christmas tree, and we gave a mathematical explanation of why this is so. It turns out that having this shape is a consequence of condition (4) of Section 3.5. In fact, we conjecture that all glycans, present and future, do satisfy (4), perhaps because of stereochemical restrictions and/or biochemical reasons. In addition, the coding reveals a rather clear-cut distinction between Bacteria and Eukaryota. The generality of our methods allows for a similar coding of future glycan DBs. Also, the coding might be of help in retrieving glycan structures in databases.

Our work suggests several questions: (a) there are “holes” in Fig. 2, e.g. around finite dimension 1 and diameters in the range 15–20, “should” there exist glycans to fill the hole? Based on their position in ${\mathscr{G}}{\mathscr{S}}$, what properties would they have (as graphs, biochemical, biological (taxonomy), etc)? (b) there are some exceptional points in Fig. 6. For example, four *EUB*-points that have dim_*f*_ < 1. What can be said about the properties of glycans coded by these four points? And what about the *BAB*-points of diameters 14 and 39 that have dim_*f*_ > 1? Or glycans coded by the 3 points with diameter ≥35? (c) More generally, is there a connection between the position of glycans in ${\mathscr{G}}{\mathscr{S}}$ and their properties?

## Electronic supplementary material

Supplementary Information
