# Supplementary material for: Finite Dimension: A Mathematical Tool to Analise Glycans
Source: Sci Rep. 2018 Mar 13;8:4426. doi: 10.1038/s41598-018-22575-4 (PMC5849774; doi:10.1038/s41598-018-22575-4)
Supplement: Supplementary file 1 — Supplementary Information [file 41598_2018_22575_MOESM1_ESM.pdf]

Supplementary Information

Finite Dimension:
A Mathematical Tool to Analise Glycans

J.M. Alonso[1,2,*], A. Arroyuelo[1], P.G. Garay[1], O.A. Martin[1], and J.A. Vila[1]

Databases

The databases *gtc, tax* and *synthetic glycogen* are available at:
https://github.com/BIOS-IMASL/finite-dimension-for-glycan-analysis

Appendix

In this Appendix we prove several claims made in sections (3.4) and (3.4.1) of the manuscript.

A1. *The point $\varphi(2,10) \in \mathcal{GS}$ is not the code of a graph.*
Proof. Suppose, for contradiction, that $\varphi(2,10) = \varphi(\Gamma)$, for some graph Γ. Thus Γ has diameter 10, and $\dim_f(\Gamma) = \ln(2)/\ln(10)$. Since such Γ would have at least 11 vertices, Theorem 5.2 in [?] applies to show that $\dim_f(\Gamma) \geq \ln(2)/\ln(3)$, a contradiction.

In what follows, $D \geq 2$ is a fixed integer.
A2. *For D-lines of triangle-free graphs, the leftmost point L_D coincides with the code of the path P_{D+1}, while the rightmost point R_D* (for arbitrary graphs) *does not exist: there are graphs of fixed diameter D whose finite dimension is as large as desired.*

Proof. We claim that, among the points of $\mathcal{GS}$ on a D-line that come from a triangle-free graph Γ, the leftmost one is given by the path P_{D+1}. Indeed, since $\dim_f(\Gamma) = \ln(N)/\ln(D)$, and $\dim_f(P_{D+1}) = \ln(\lceil (D+1)/2 \rceil)/\ln(D)$, the claim is equivalent to $\lceil (D+1)/2 \rceil \leq N$. If $m := |V(\Gamma)|$, then $m \geq D+1$, since Γ has diameter D. On the other hand, $\lceil m/2 \rceil \leq N$, because Γ is triangle-free. Hence, $N \geq \lceil (D+1)/2 \rceil$, as was to be proved.

We prove the second claim. Given D, n, consider the tree $\Gamma := P_D \cup St_n$, where one of the end-vertices of P_D is identified to the central vertex of St_n. Note that the central vertex has degree n. Then $\gamma(\Gamma)$ lies on the D-line, and $R_D \geq \dim_f(\Gamma) = \ln(n - 1 + \lceil (D-1)/2 \rceil)/\ln(D) \to \infty$, when $n \to \infty$, as desired. The proof is complete.

A3. *The rightmost point of D-lines of chemical graphs is finite, but tends to infinity with D.*

Proof. Let Γ be a chemical graph of diameter D. Then Γ has no more than $1 + 2(3^D - 1)$ vertices (see for instance [1]). It follows that there are only finitely many chemical graphs of any given diameter and, hence, that $R_D < \infty$.

To prove the last claim, we construct examples of graphs whose $\dim_f$ tends to infinity with D. All our examples are subgraphs of T, an infinite tree with all vertices of degree 4. A concise way to define T is to identify it to the Cayley graph of F_2, the free (non-abelian) group on two generators x, y, with respect to the generating set $\{x^{\pm 1}, y^{\pm 1}\}$; T is a regular infinite tree of degree 4.

Suppose $D = 2n$ is even. Let $B_n \subset T$ denote the ball of radius n centered at, say, the identity e. By "ball" we mean, in this case, the complete subgraph generated by all vertices at distance $\leq n$ from e. There are $4 \cdot 3^{n-1}$ vertices of degree 1 in B_n (the "boundary" of the ball), all other vertices have degree 4. To compute $\dim_f(B_n) = \ln(N)/\ln(D)$, for $n = 2k+1$, note that (for $k > 0$) $N = 4(\sum_{i=0}^{k} 3^{2i}) > 4(3^{2k}) > 3^{D/2}$, hence $\dim_f(B_n) \to \infty$ when $D \to \infty$, as claimed. The same is true when $n = 2k$ is even, since in this case $N = 1 + 4(\sum_{i=0}^{k-1} 3^{2i+1}) > 4(3^{2k-1}) > 3^{D/2}$.

For $D = 2n + 1$ odd, one can consider maximal subgraphs of T of diameter D; these can also be described as follows. Take the ball B_n as before, and notice that it consists of 4 branches, say $G_1, \ldots, G_4$, joined at e. Let V' denote the set of 3^{n-1} vertices of G_1 at distance n from e. In T, there is a set V'' of 3^n vertices that satisfy these two conditions: They are at distance $n + 1$ from e, and at distance 1 from V'. Let Γ denote the complete subgraph of T that contains $B_n \cup V''$: It is connected, has diameter D, $3(3^{n-1}) + 3^n$ vertices of degree 1 (the boundary of Γ), and the rest of the vertices have degree 4. The covering number N is larger than the covering number N' of B_n and, as before $N' > 3(3^{n-1})$, so that $N > N' > 3(3^{n-1}) = 3^{(D-1)/2}$. This implies that $\dim_f(\Gamma) \to \infty$ when $D \to \infty$. The proof is complete.

References

[1] Miller, M. and Širáň, J. Moore graphs and beyond: A survey of the degree/diameter problem. *The Electronic Journal of Combinatorics*, **20(2)**, 1-92, (2013).
